# Comparison of Ultrasonic Scalpel versus Conventional Techniques in Open Gastrectomy for Gastric Carcinoma Patients: A Systematic Review and Meta-Analysis

**DOI:** 10.1371/journal.pone.0103330

**Published:** 2014-07-31

**Authors:** Xiao-Long Chen, Xin-Zu Chen, Zheng-Hao Lu, Li Wang, Kun Yang, Jian-Kun Hu, Bo Zhang, Zhi-Xin Chen, Jia-Ping Chen, Zong-Guang Zhou

**Affiliations:** 1 Department of Gastrointestinal Surgery, West China Hospital, Sichuan University, Chengdu, Sichuan Province, China; 2 Chinese Cochrane Center, West China Hospital, Sichuan University, Chengdu, Sichuan Province, China; Cardiff University, United Kingdom

## Abstract

**Objectives:**

To compare surgical efficacy and postoperative recovery of ultrasonic scalpel (USS) with conventional techniques for the resection of gastric carcinoma.

**Methods:**

A systematic search of major medical databases (PubMed, Embase, CCRT and CNKI) was conducted. Both randomized and non-randomized controlled trials (RCTs and nRCTs) were considered eligible. Operation time (OT), intraoperative blood loss (BL) and postoperative complications (POC) rates as well as postoperative hospitalization days, number of dissected lymph nodes, abdominal drainage volume and time for recovery of gastrointestinal functions were synthesized and compared.

**Results:**

Nineteen studies were included (7 RCTs and 12 nRCTs), in which 1930 patients were enrolled totally (946 in the USS group and 984 in the conventional group). Monopolar electrocautery and ligation were used as the conventional methods. Comparative meta-analysis showed perioperative outcomes were significantly improved using USS compared with conventional surgical instrumentation. OT was reduced from a weighted mean of 185.3 min in the conventional group to 151.0 min in the USS group (MD = −33.30, 95% CI [−41.75, −24.86], p<0.001) and intraoperative BL was decreased from a weighted mean of 217.9 ml in the conventional group to 111.6 ml in the USS group (MD = −113.42, 95% CI [−142.05, −84.79], p<0.001). Results from RCTs subgroup were consistent with those from nRCTs subgroup. The weighted cumulative risk of POC accounted for 8.9% (0%–25%) and 12.9% (5.5%–45%) in the USS and conventional groups, respectively. Pooled estimated results from nRCTs (OR = 0.54, 95% CI [0.27, 1.06], p = 0.07) and RCTs (RR = 0.75, 95% CI [0.44, 1.26], p = 0.27) showed no significant difference between the USS and control groups. Analysis of secondary outcomes showed the improvements of the USS group over control group regarding the number of dissected lymph nodes, postoperative hospitalization days, abdominal drainage volume and time for recovery of gastrointestinal functions.

**Conclusion:**

Compared with conventional electrosurgery, the USS is a safe and effective technique with more short-term advantages in open surgery for gastric cancer.

## Introduction

Gastric carcinoma (GC) is one of the most common malignancies worldwide with high incidence and cancer-related mortality, especially in Asia [1]–[3]. To date, surgery is still the important therapeutic strategy for GC [4]–[7]. Standardized radical gastrectomy plus lymphadenectomy is the vital procedure for GC, although with many operative difficulties [8]. A number of novel surgical tools are emerging, with the aim of reducing operation time, rates of surgical injury, and postoperative complication. Among them, ultrasonic scalpel (USS) has been widely used in many kinds of surgery like cholecystectomy, colectomy, and glossectomy [8]–[11]. With high-frequency sonic wave vibration, approximately 55,000 Hz, the USS can facilitate target tissues concretion and degeneration to accomplish hemostasis [12]–[13]. Commonly, the USS can cut off and seal the vessels (including lymphatics) with the diameter less than 5 mm. Compared with conventional monopolar electrocautery or silk thread ligation, the USS is capable of simplifying surgical procedures and reducing operation time by one-step cutting and coagulation. In view of excellent hemostasis with slight damages to the target tissues and inconspicuous thermal effect to the tissues around the scalpel, the USS has been widely used in laparoscopic and open gastrectomy plus lymphadenectomy for GC patients in the world.

Although numerous studies had compared the USS with conventional techniques for GC, they were mainly retrospective or population limited and thus insufficient to evaluate the surgical outcomes among these different techniques. Therefore, we performed a systematic review and meta-analysis to compare the surgical efficacy and postoperative recovery of USS with those of conventional techniques in open gastrectomy plus lymphadenectomy for GC patients.

## Methods

This systematic review and meta-analysis was conducted in accordance with the PRISMA statement [14]. No protocol was registered.

### Search strategy

Published randomized controlled trials (RCTs) and controlled clinical trials with language restriction to English or Chinese in the following electronic databases: Cochrane Central Register of Controlled Trials (CCRT) (up to September 12, 2012), PubMed (up to September 28, 2012), Embase (up to September 12, 2012) and China National Knowledge infrastructure (CNKI) (up to October 7, 2012) were searched. The literature search in PubMed was carried out following the strategy shown in Table 1. The search strategy was also referred in other electronic databases.

**Table 1:** Search strategy used in PubMed database.

Search number	Search query	Search fields
#1	gastric OR stomach	All fields
#2	cancer OR carcinoma OR tumor OR tumour OR neoplasm	All fields
#3	#1 AND #2	
#4	(stomach neoplasm) OR (gastric cancer)	MeSH Terms
#5	#3 OR #4	
#6	harmonic OR ultrasound OR ultrasonic OR ultrasonically OR CUSA	All fields
#7	dissector OR scalpel OR knife OR shear	All fields
#8	#6 AND #7	
#9	(ultrasonic surgical procedures) OR (high-energy shock waves)	MeSH Terms
#10	#8 OR #9	
#11	#5 AND #10	

### Inclusion and exclusion criteria

Only studies comparing the USS with conventional techniques in gastrectomy were included in this analysis. Both RCTs and non-RCTs (nRCTs) were eligible. The USS and conventional techniques should be used in the same procedures during operations. Studies were excluded if (1) they were irrelevant to gastric cancer but focused on other cancers, like colon cancer; (2) there was only the USS group but no control group reported; (3) the outcomes of interest were not reported; (4) there was considerable overlap between authors, centers or patient cohorts; and (5) the USS combined with other methods was compared with the USS alone.

### Selection, assessment, and data extraction

Two independent authors (Chen XL and Lu ZH) assessed the titles and abstracts of all the studies identified by the initial search to exclude the obviously irrelevant studies, such as those on colon cancer and prostatic tumor, and those with only the USS group but without control group. After that, they obtained the full texts of all potentially relevant studies and also those with unclear methodology for further selection to exclude inappropriate studies, such as those reporting on the USS and other methods versus those reporting on the USS alone. Subsequently, the qualities of RCTs and nRCTs remaining were assessed by two authors using the Jadad Scale [15] and Newcastle-Ottawa Scale (NOS) [16], respectively. Primary outcome measures included the following: 1) operation time (OT), 2) postoperative complications (POC), and 3) intraoperative blood loss (BL). Secondary outcome measures were as follows: 4) number of dissected lymph nodes (NDLN), 5) postoperative hospitalization days (POHD), 6) number of transfusion patients (NTP), 7) abdominal drainage (AD), and 8) gastrointestinal function recovery days (GIFRD). Data on outcome measures and sample details were also extracted. At each level of screening, agreement between two authors was assessed. Any disagreements in study assessment and data collection were discussed and resolved by a third party (Hu JK and Chen XZ) as the referees.

### Statistical analysis

The statistical analysis was performed using Reviewer Manager (RevMan) software version 5.0 (provided by the Cochrane Collaboration). Data were analyzed for odds ratio (OR) and risk ratio (RR) in the cases of dichotomous variables with the Mantel-Haenszel (M-H) test and for mean difference (MD) in continuous variables with inverse variance (IV) test. The 95% confidence intervals (CIs) of MD, RR, and OR were also calculated. A two-sided p value less than 0.05 was considered as a significant difference. Between-trial heterogeneity was evaluated using the chi-square test; p value less than 0.1 was considered as a significant heterogeneity [17]. Provided that heterogeneity existed, the random effect model was used for meta-analysis; otherwise, the fixed effects model was applied. To compare the average level of different outcomes, the weighted cumulative mean (WCM) and risk (WCR) were also calculated. The difference of means and risks was tested using the independent sample T test if normal distribution and equal variances existed; otherwise, rank-sum test with Mann-Whitney U test was used.

## Results

### Literature search and selection

**Figure 1:**
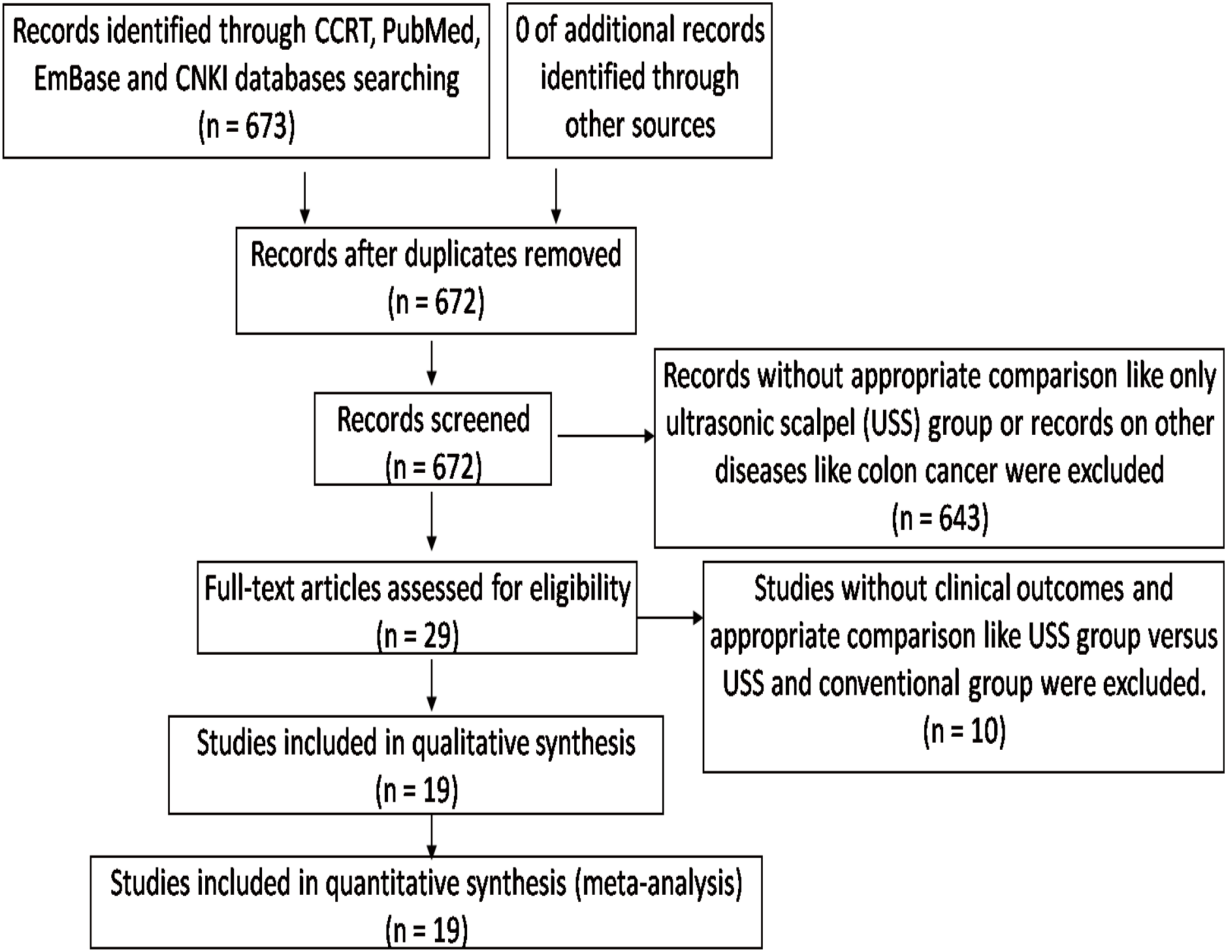
Literature search and selection procedure.

The literature search and selection procedures were shown in Figure 1. Two articles reported the same population [18]–[19]. Finally, 19 studies (7 RCTs and 12 nRCTs) reporting open gastrectomy were eligible for analysis, and 1930 patients (946 in the USS group and 984 in the conventional group) were included [18]–[37] (Table 2). Respective scale dimensions for each score after the evaluation by the Jadad Scale and the NOS were shown in Table 3. The sample size of each study ranged from 40 to 296. There were no significant differences in the baselines between the USS and the control groups in these studies, as reported. In the included studies, the USS was compared with conventional techniques–monopolar electrocautery and ligation by silk thread. The types of USS were mainly harmonic focus or GEN300/STM (5 mm) from Johnson-Johnson Company from USA, Olympus from Japan and SONICA from Germany. Of the 19 studies, only one had reported perioperative mortality, which showed one patient in the USS group and one in the conventional group died because of disease progression [21]. Other studies had no reports about perioperative death. The WCM and WCR of all outcomes in the USS and conventional groups were shown in Table 4. Relevant characteristics of all Chinese studies included were tabulated for ease of the references in Table 5.

**Table 2:** Summary information of included studies.

Studies	Demographic data	Intervention	JS/NOS
Inoue K, et al [37],2012, Japan, RCT	30 patients in each group with resectable GCwere underwent open gastrectomy with D0–D2dissection. Combined resections includinggallbladder, spleen and spleen pancreatic tailwere performed in 6 patients in each group.	USS group: harmonic focus USS for ≤5 mmvessels and lymphatics and electrocautery for dissectionof avascular planes, minute vessels and lymphatics.Conventional group: only electrocautery and ligation with silk thread.	1
Tsimoyiannis EC,et al [20], 2001,Greece, RCT	20 patients in each group were underwent opentotal or subtotal gastrectomy with D2dissection. Spleen was resected in carcinomaof cardia, fundus and upper part of the corpus.	USS group: ultracision harmonic shears of 10 mmin all steps of dissection, hemoclips or ligations forblood vessels more than 3 mm. Conventional group: monopolar electrosurgeryfor cutting and coagulation, hemoclips or ligations to obstruct the vessels.	1
Chen CP [25],2012, China,RCT	60 patients in each group were underwentgastrectomy with D2 dissection.	USS group: GEN300/STM (5 mm) USS.Conventional group: unclear.	2
Liu L, et al [26],2010, China,RCT	19 patients in USS group and 21 inconventional group were underwent distalgastrectomy with D2 dissection.	USS group: GEN300/STM (5 mm) USS.Conventional group: monopolar electrocautery was used inall the course of operations.	2
Xu L, et al [34],2010, China,RCT	23 patients in USS group and 19 inconventional group underwent gastrectomywith D2 dissection.	USS group: Ethicon USS.Conventional group: monopolar electrocautery and ligation.	1
Zhang ZY [27],2012, China,RCT	50 patients in USS group and 48 inconventional group were underwent radicalgastrectomy.	USS group: SONACA150 USS for ≤5 mm vessels andGN300 electrocautery. Conventional group: GN 300electrocautery and ligation.	3
Lu WQ, et al [30],2008, China,RCT	26 patients in USS group and 23 inconventional group were underwentgastrectomy with D2 dissection.	USS group: GEN 300 STM 5 mm.Conventional group: GD 350-Dmonopolar electrocautery.	1
Mohri Y, et al[21], 2007, Japan,nRCT	26 patients in each group with ≥7 cmGC were underwent primary open totalor distal gastrectomy with D1–D2dissection. 26 patients wereunderwent adjacent organ resection.	USS group: ultracision harmonic shears used in all stepsof dissection to seal lymphatic tissue and ligate theperigastric vessels. Left and right gastroepiploic andgastric vessels were ligated. Conventional group: onlymonopolar electrosurgery for cutting and coagulation.The blood vessels and main lymphatic vessels were ligated.	8
Li G, et al [19],2010, China,nRCT	97 in USS and 122 in conventionalgroup were underwent gastrectomy withD2 dissection.	USS group: GEN 300/STM (5 mm) USS.Conventional group: GD-350D monopolar electrocautery.	9
Wei ZM, et al[22], 2010,China, nRCT	34 patients in USS group and 38 inconventional group, who were morethan 60 years were underwentgastrectomy with D2 dissection.	USS group: ultrasonic harmonic scalpel for ≤5 mmvessels and ligation for >5 mm vessels. Conventionalgroup: monopolar electrocautery and other conventionaltechniques for division, ligation and cutting.	7
Tu JC, et al [23],2010, China,nRCT	156 patients in USS group and 140 inconventional group were underwentstandard distal gastrectomy withD2+No.14V dissection.	USS group: GEN300 USS was used for vessels exceptleft and right gastric, right gastroepiploic vessels.Conventional group: monopolar electrocautery and ligation	8
Fu YM [24],2011, China,nRCT	70 patients in each group wereunderwent gastrectomy with D2dissection.	USS group: USS resources unclear. Dissected allLN then cut off stomach and duodenum. Conventionalgroup: dissected NO.6 and NO12a LN then cut offstomach and duodenum, then dissected other LN.	8
Li P, et al [28],2011, China,nRCT	111 patients in USS group and 120 inconventional group underwentgastrectomy with D2 dissection.	USS group: GEN 300/STM (5 mm) USS.Conventional group: GD-350D monopolarelectrocautery and ligation in all course operation.	9
Tu XH, et al [29],2009, China,nRCT	42 patients in USS group and 54 inconventional group were underwenttotal and distal gastrectomy with D2dissection.	USS group: harmonic wave TM and harmonicTM 300 (CEN 04). Conventional group: monopolar electrocautery.	9
Chen Z, et al [31],2009, China,nRCT	21 patients in USS group and 25 inconventional group were underwentradical gastrectomy.	USS group: USS from Harmonic Ethicon Endo.Conventional group: Force FXTM-8C.	7
Li ZR, et al [32],2009, China,nRCT	49 patients in USS group and 56 inconventional group were underwentgastrectomy with D2+NO14v LNdissection.	USS group: GEN 300/STM (5 mm).Conventional group: GD-3502D monopolar electrocautery	9
Lin YH, et al[33], 2011,China, nRCT	35 patients in USS group and 28patients in conventional groupwere underwent gastrectomy withD2+NO.14v LN dissection.	USS group: GEN 300 USS and monopolar electrocautery.Conventional group: monopolar electrocautery and ligation.	9
Shi YF, et al [35],2012, China,nRCT	30 patients in USS and 30 inconventional group wereunderwent gastrectomywith D2 dissection.	USS group: GEN300 USS alone for all vessels but withligation for left and right gastroepiploic and gastric vessels.Conventional group: GD-350D monopolar electrocautery and ligation.	9
Song XP, et al[36], 2011,China, nRCT	47 patients in USS group and 54 inconventional group wereunderwent gastrectomy with D2dissection.	USS group: OLYMPUS USSfor ≤3 mm vessels without ligation.Conventional group: monopolar electrocautery.	8

Abbreviations: GC: gastric carcinoma; USS: ultrasonic scalpel; RCT: randomized controlled trials; nRCT: non-randomized controlled trials; LN: lymph nodes; JS: Jadad Scale; NOS: Newcastle-Ottawa Scale; JS was for RCTs and NOS for nRCTs.

**Table 3:** Respective scale dimensions for each score of Jadad Scale and Newcastle-Ottawa Scale.

Study type	Number of study	Evaluation	Scores	Included studies	Percentage
RCT	7	Jadad Scale	1	4	57%
			2	2	29%
			3	1	14%
nRCT	12	Newcastle-Ottawa Scale	7	2	17%
			8	4	33%
			9	6	50%

Abbreviations: RCT: randomized controlled trials; nRCT: non-randomized controlled trials.

**Table 4:** Details of weighted cumulative mean and risk of outcomes in USS group and conventional group.

Outcomes	Study	References	USS group		Conventional group		Weighted mean difference(95% CI)	P value
	counts		Patients	WCM/WCR	Patients	WCM/WCR		
OT (min)	14	[19]–[23], [25]–[29], [31], [34]–[36]	736	151.0	777	185.3	−33.30 (−41.75, –24.86)	<0.001
POC (n)	9	[19]–[21], [27], [29]–[31], [36]–[37]	359	0.089	402	0.129	Not applicable	Not applicable
BL (ml)	15	[19]–[23], [25]–[29], [31], [33]–[36]	771	111.6	805	217.9	−113.42 (−142.05, –84.79)	<0.001
NDLN (n)	13	[19, 22–25, 27–31, 33–34,36–36]	977	20.2	903	18.9	2.48 (1.02, 3.94)	<0.001
POHD(days)	3	[20], [22], [29]	96	11.3	112	13.1	−1.69 (−2.27, –1.12)	<0.001
NTP (n)	3	[20]–[21], [37]	76	0.18	76	0.36	Not applicable	Not applicable
AD (ml)	7	[22]–[24], [26]–[27], [32]–[33]	413	199.2	401	302.8	−96.67 (–119.26, –74.09)	<0.001
GIFRD (days)	6	[19], [25], [28]–[29], [34]–[35]	363	3.1	405	4.0	−0.94 (–1.20, –0.64)	<0.001

Abbreviations: USS: ultrasonic scalpel; OT: operation time; POC: postoperative complications; BL: blood loss in operations; NDLN: number of dissected lymph nodes; POHD: postoperative hospitalization days; NTP: number of transfusion patients; AD: abdominal drainage; GIFRD: gastrointestinal function recovery days; WCM: weighted cumulative mean; WCR: weighted cumulative risk.

**Table 5:** Characteristics of included Chinese studies for ease of reference.

First author	Year	Type	Surgery	Sample size	Operation time	Postoperative complications	Blood loss in operation	Number of dissected lymph nodes	Postoperative hospitalization days	Abdominal drainage	Gastrointestinal function recovery days
Chen CP [25]	2012	RCT	USS	60	182.5±47.3		101.6±72.1	21.2±6.7			2.8±0.6
			Conventional	60	201.4±51.2		193.7±68.1	22.3±7.1			3.9±0.7
Liu L [26]	2010	RCT	USS	19	110±15		220±20			165±20	
			Conventional	21	165±20		350±30			250±15	
Xu L [34]	2010	RCT	USS	23	171.2±52.5		97.3±74.1	24.1±4.7			3.0±0.5
			Conventional	19	202.8±47.9		186.1±67.4	23.3±4.1			3.9±0.7
Zhang ZY [27]	2012	RCT	USS	50	131±17	8	57±35	15±4		105±31	
			Conventional	48	156±20	9	105±50	15±3		169±29	
Lu WQ [30]	2008	RCT	USS	26		0		21		226	
			Conventional	23		2		20		712	
Yin B [18]*	2011	nRCT	USS	97	160±35		93±40				2.9±1.7
			Conventional	122	202±41		152±67				3.9±1.6
Li G [19]*	2010	nRCT	USS	97	160±35	6	93±40	16±3.4			2.9±1.7
			Conventional	122	202±41	7	152±67	13.1±3.3			3.9±1.6
Wei ZM [22]	2010	nRCT	USS	34	172.1±18.2		105.2±24.3	27.3±4.4	12.1±1.2	561.9±85.2	
			Conventional	38	224.3±23.5		208.6±52.4	21.3±6.8	13.8±1.6	591.9±105.6	
Tu JC [23]	2010	nRCT	USS	156	110±35		110±60	24±5		180±60	
			Conventional	140	135±40		140±75	23±6		270±90	
Fu YM [24]	2011	nRCT	USS	70				26±4		178±54	
			Conventional	70				23±3		280±65	
Li P [28]	2011	nRCT	USS	111	170.2±52.5		97.5±74.1	24.2±4.7			3.1±0.5
			Conventional	120	202.5±47.9		186.2±67.4	23.4±4.1			3.8±0.7
Tu XH [29]	2009	nRCT	USS	42	128.2±34.1	1	124.2±39.4	23.5±5.1	11.5±2.9	173.9±30.2	4.1±1.1
			Conventional	54	165.6±40.5	3	274.6±64.6	21.5±5.5	12.9±3.6	289.8±46.1	4.5±1.4
Chen Z [31]	2009	nRCT	USS	21	125±21	0	50±15	25±11			
			Conventional	25	145±29	2	72±28	23±14			
Li ZR [32]	2009	nRCT	USS	49						169.0±56.6	
			Conventional	56						358.0±125.6	
Lin YH [33]	2011	nRCT	USS	35			50.1±20.7	32.1±4.6		170.2±28.4	
			Conventional	28			171.4±30.4	20.2±5.1		289.7±46.2	
Shi YF [35]	2012	nRCT	USS	30	175.6±45.6		95.4±45.3				3.0±0.5
			Conventional	30	210.5±50.4		185.5±60.8				4.5±1.0
Song XP [36]	2011	nRCT	USS	47	150±36	3	115±96	20.1±4.4			
			Conventional	54	197±62	8	426±115	18.9±4.6			

Abbreviations: USS: ultrasonic scalpel. RCT: randomized controlled trials; nRCT: non-randomized controlled trials. *: both studies were based on the same population.

### Primary outcomes

#### Operation time

**Figure 2:**
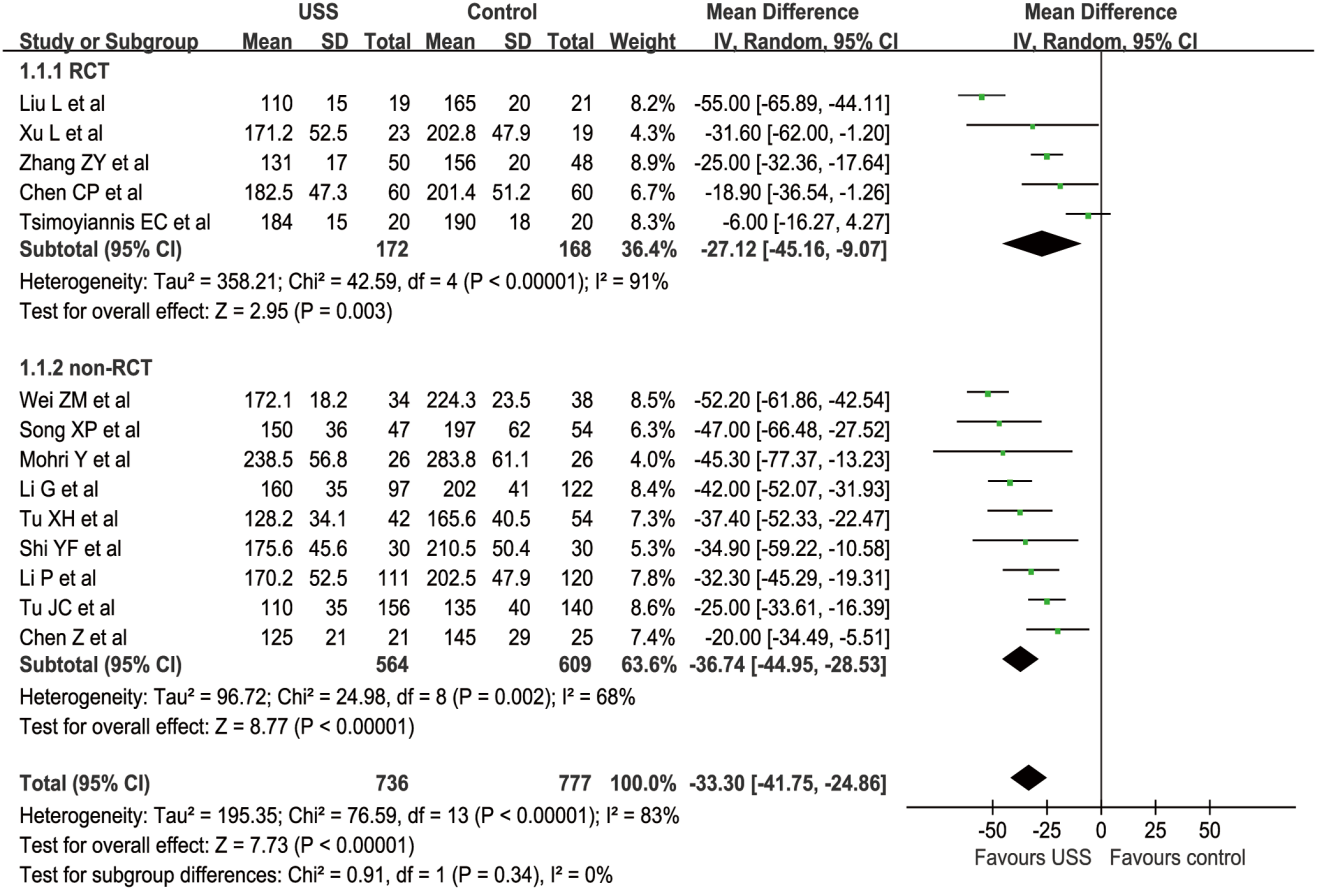
Forest plot of operation time.

**Figure 3:**
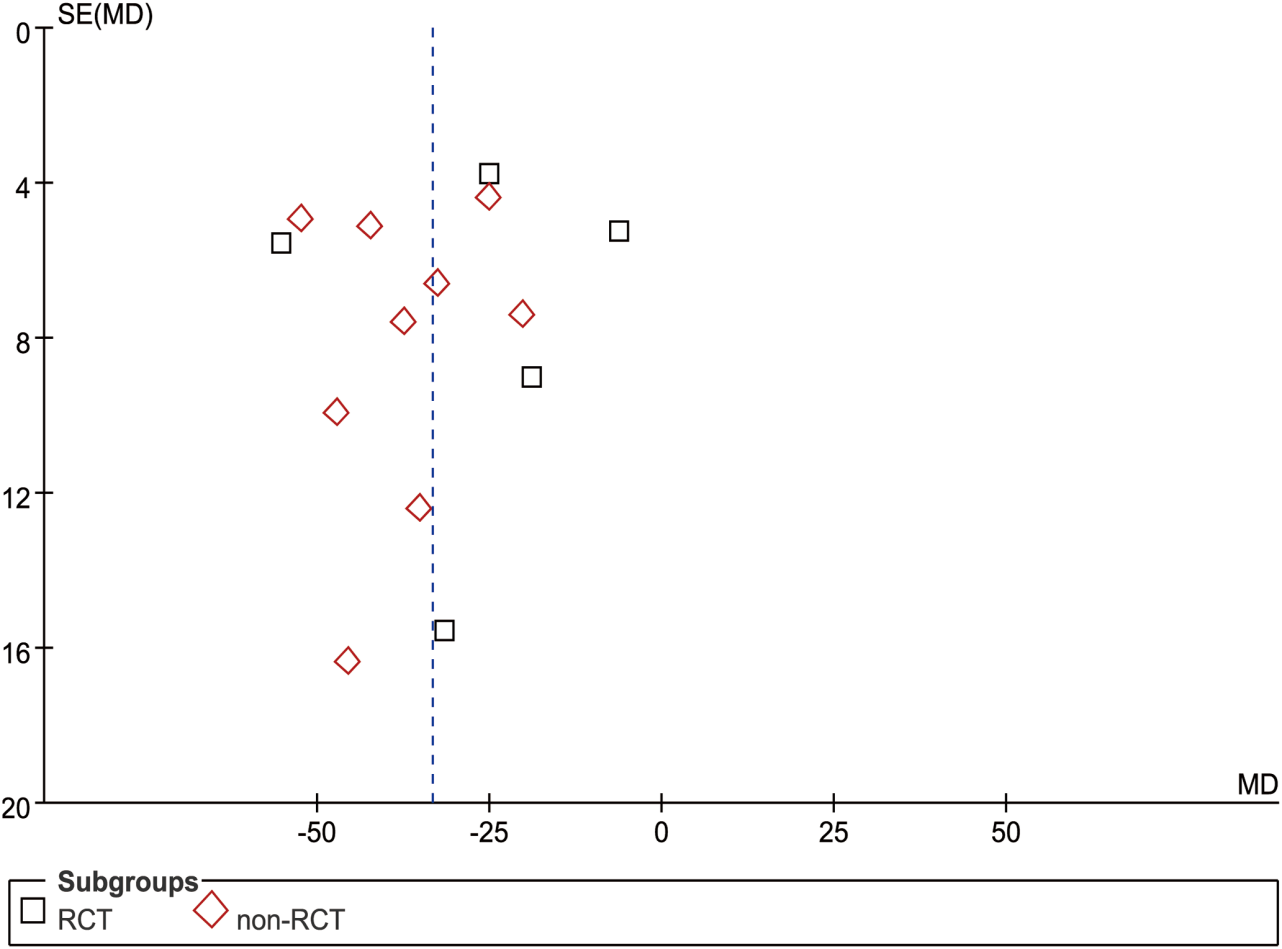
Funnel plot of operation time.

Fourteen studies (5 RCTs and 9 nRCTs) reported OT from a mean of 110 to 238.5 min in the USS group and 135 to 283.8 min in the conventional group [19]–[23], [25]–[29], [31], [34]–[36]. The WCM was 151.0 min in the USS group and 185.3 min in the conventional group. Meta-analysis indicated significantly less OT in the USS group than that in the conventional group in both RCT (MD = −27.12, 95% CI [−45.16, −9.07], p = 0.003) and nRCT (MD = −36.74, 95% CI [−44.95, −28.53], p<0.001) subgroup sensitivity analysis. Generally, the USS group could decrease by approximately half an hour of OT compared with the control group (MD = −33.30, 95% CI [−41.75, −24.86], p<0.001) (Figure 2). The funnel plot showed a symmetrical distribution of included studies (Figure 3).

#### Postoperative complications

**Figure 4:**
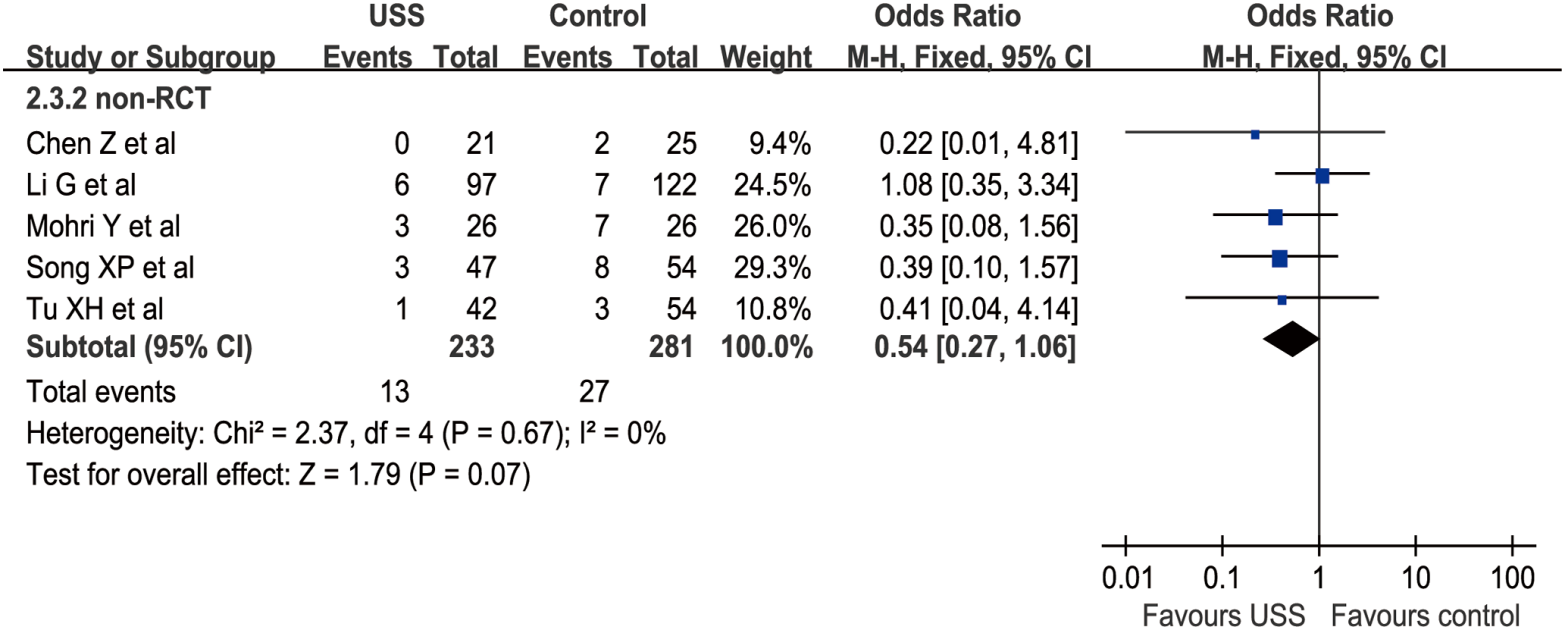
Forest plot of postoperative complications in nRCTs subgroup.

**Figure 5:**
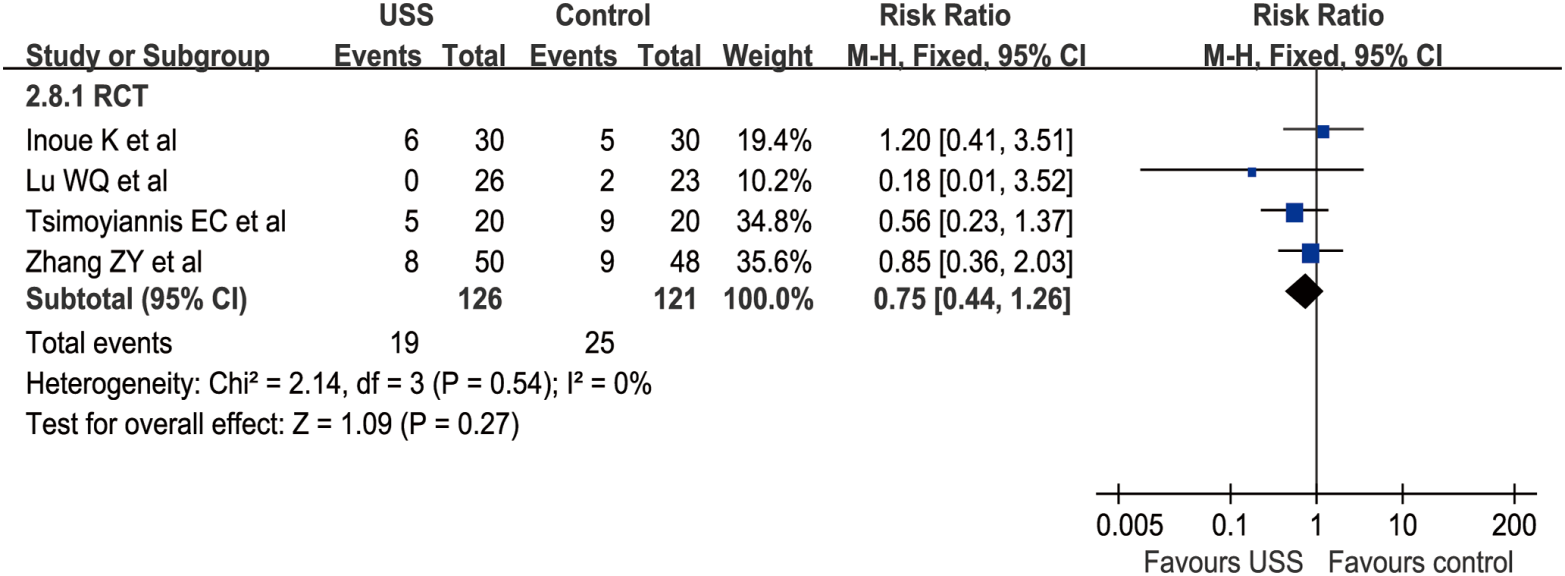
Forest plot of postoperative complications in RCTs subgroup.

Nine studies (4 RCTs and 5 nRCTs) described POC from 0% to 25% in the USS group and 5.5% to 45% in the conventional group [19]–[21], [27], [29]–[31], [36]–[37]. The main complications were anastomosis leakage, lymphatic leakage, pancreatic leakage, incision infection, gastroparesis, cardiac ischemia, and respiratory failure. The WCR was 0.089 versus 0.129 in the USS and conventional groups, respectively. The total effect was not calculated due to the difference between RR in RCTs and OR in nRCTs. Meta-analysis revealed no significant difference between the USS group and the control group in the nRCT subgroup (OR = 0.54, 95% CI [0.27, 1.06], p = 0.07) (Figure 4) and RCT subgroup (RR = 0.75, 95% CI [0.44, 1.26], p = 0.27) (Figure 5). Attributable to the limited number of RCTs and nRCTs, the funnel plots of both subgroups were not applied.

#### Blood loss in operations

**Figure 6:**
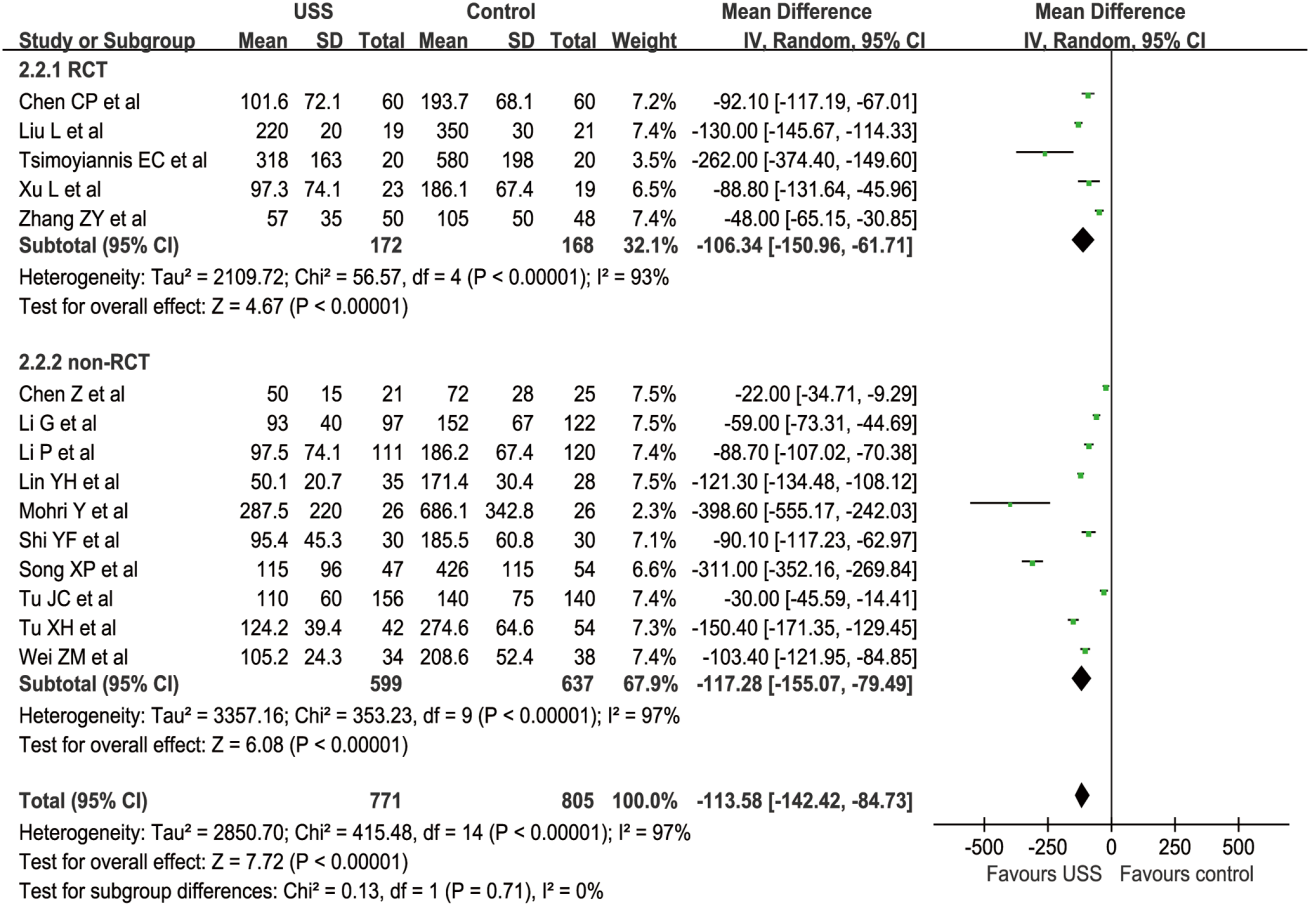
Forest plot of blood loss in operation.

**Figure 7:**
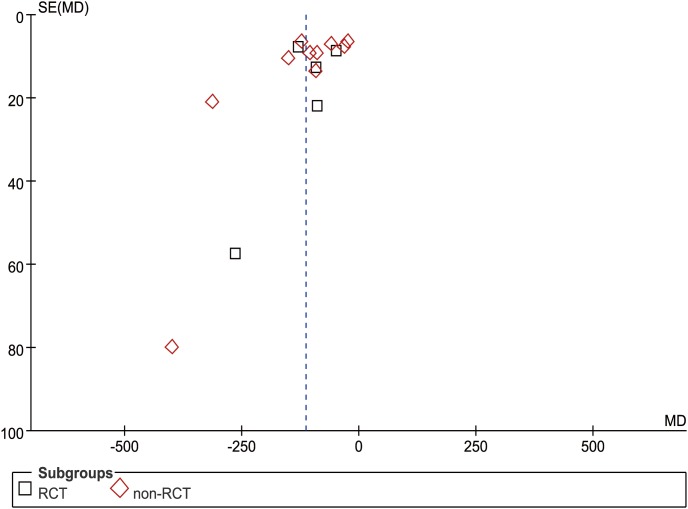
Funnel plot of blood loss in operation.

In total, 15 studies (5 RCTs and 10 nRCTs) reported BL from a mean of 50 to 287.5 ml in the USS group and 72 to 686.1 ml in the conventional group [19]–[23], [25]–[29], [31], [33]–[36]. The WCM was 111.6 ml in the USS group and 217.9 ml in the control group. BL was significantly less in the USS group than in the control group in both RCT (MD = −106.34, 95% CI [−150.96, −61.71], p<0.001) and nRCT (MD = −117.06, 95% CI [−154.46, −79.66], p<0.001) subgroup sensitivity analysis. Generally, compared with the control group, the USS group could diminish by approximately 100 ml of hemorrhagic volume (MD = −113.42, 95% CI [−142.05, −84.79], p<0.001) (Figure 6). Additionally, some studies reported blood loss using different criteria (“gram” or “milliliter”). Because blood density was close to 1 g/ml [38], we simply changed the unit “gram” into “milliliter” without changing the value. Although the funnel plot showed an asymmetrical distribution with more studies located at the right part of the middle line, the difference was still significant. Therefore, the results were proved to be reliable on the contrary (Figure 7).

### Secondary outcomes

#### Number of dissected lymph nodes

**Figure 8:**
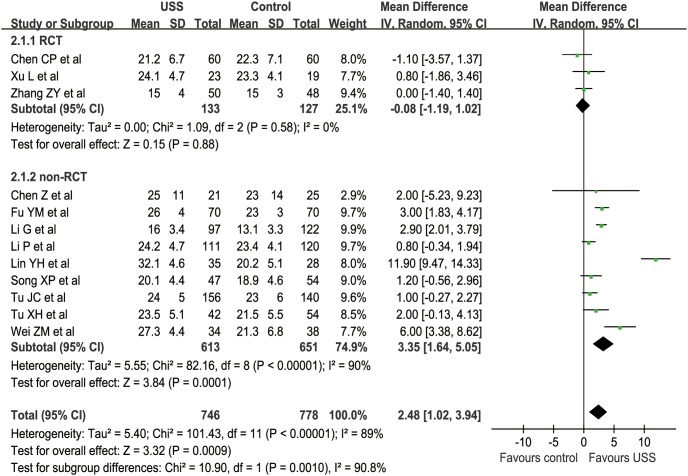
Forest plot of number of dissected lymph nodes.

**Figure 9:**
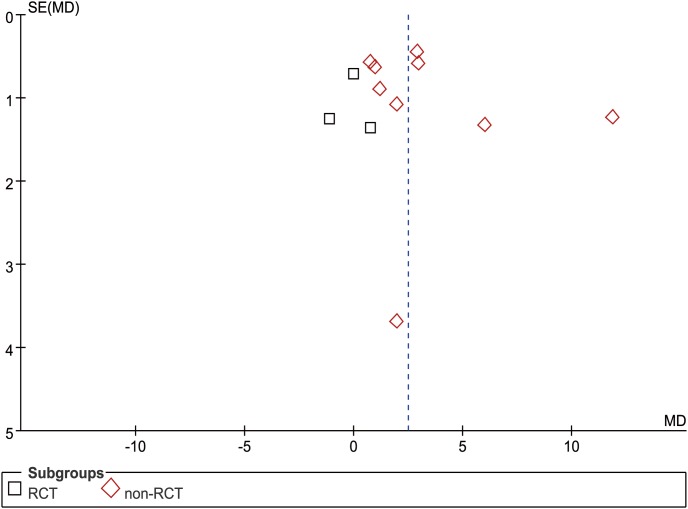
Funnel plot of number of dissected lymph nodes.

The WCM of NDLN from 13 studies was 22.6 in the USS group and 20.4 in the control group [19], [22]–[25], [27]–[31], [33]–[34], [36]. However, only 12 studies (3 RCTs and 9 nRCTs) simultaneously reported the mean value (from 13.1 to 32.1) and standard deviation of dissected lymph nodes (LNs) [19], [22]–[25], [27]–[29], [31], [33]–[34], [36]. Two studies demonstrated D0–D2 and D1–D2, respectively [21], [37]. Other studies detailed at least D2 or radical dissection. Only one study did not reach at least 15 dissected LNs as recommended in the National Comprehensive Cancer Network (NCCN) [39], in which the mean value of the harvested LNs of the conventional group was 13.1 [19]. Another study did not reach at least 16 LNs as recommended in the Japanese Gastric Cancer Association (JGCA) guidelines [40], with average of 15 harvested LNs [27]. Meta-analysis revealed that more LNs were dissected in the USS group than in the control group in nRCT subgroup (MD = 3.35, 95% CI [1.64, 5.05], p<0.001) but not in the RCT subgroup (MD = −0.08, 95% CI [−1.19, 1.02], p = 0.88). For a total effect, the USS group could dissect about two LNs more than the conventional group (MD = 2.48, 95% CI [1.02, 3.94], p<0.001) (Figure 8). Although the funnel plot showed an asymmetrical distribution with more studies located at the left part of the middle line, the difference was still significant. Hence, the results were proved to be reliable on the contrary (Figure 9).

#### Postoperative hospitalization days

**Figure 10:**
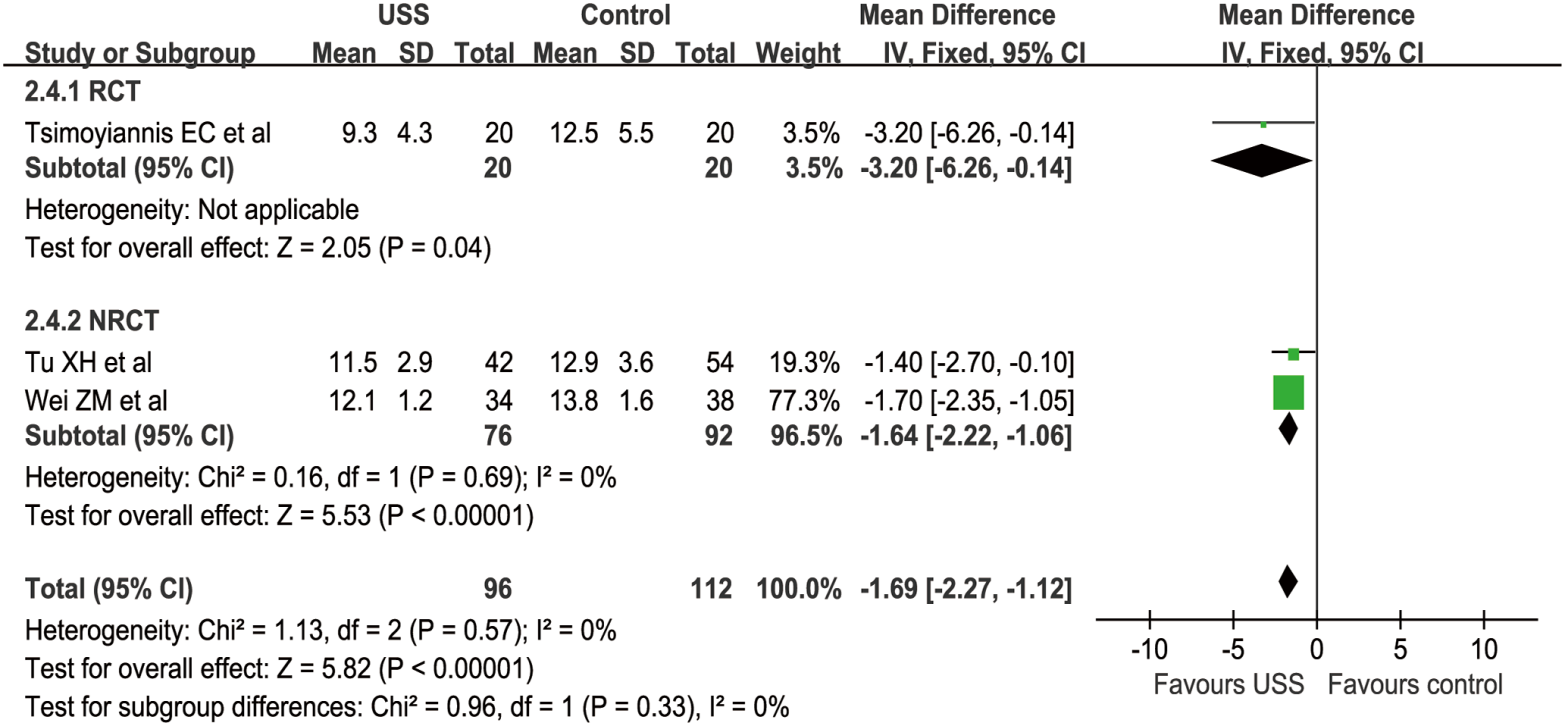
Forest plot of postoperative hospitalization days.

Three studies (1 RCT and 2 nRCTs) reported POHD from a mean of 9.3 to 13.8 days [20], [22], [29]. The WCM was 11.3 and 13.1 days in the USS and control groups, respectively. Meta-analysis was not suitable in the RCT subgroup because there was only one study in this group, which reported a mean of 9.3 days compared with 12.5 days in the USS and conventional groups, respectively (MD = −3.2, 95% CI [−6.26, −0.14], p = 0.04). In nRCT subgroup, fewer POHD were found in the USS group than in the control group (MD = −1.64, 95% CI [−2.22, −1.06], p<0.001). For overall effect, patients in the USS group had obviously shorter POHD than those in the conventional group (MD = −1.69, 95% CI [−2.27, −1.12], p<0.001) (Figure 10). The funnel plot was not applied because of the limited number of included studies.

#### Number of transfusion patients

**Figure 11:**
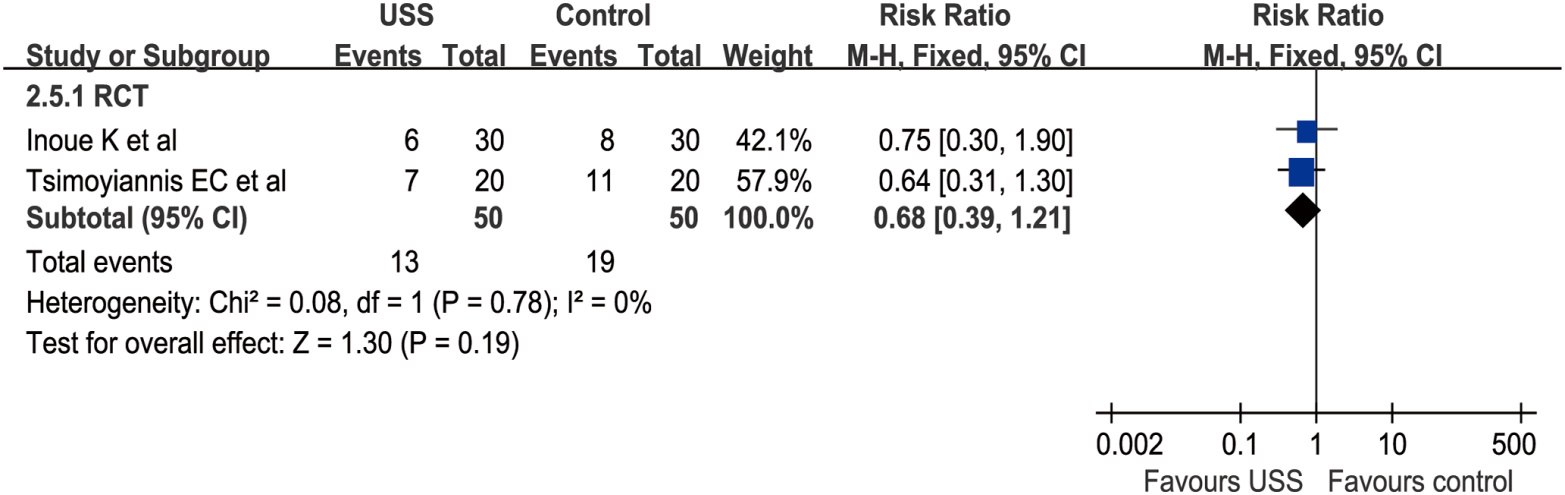
Forest plot of number of transfused patients in RCT subgroup.

Three studies (2 RCTs and 1 nRCT) reported NTP from 1 to 11 patients [20]–[21], [37]. The WCR were 0.18 and 0.36 in the USS and conventional groups, respectively. Similarly, meta-analysis was not applied in the nRCT group because there was only one study, which reported 1 patient in the USS group versus 8 patients in the conventional group (OR = 0.09, 95% CI [0.01, 0.78], p = 0.03). In RCT subgroup, meta-analysis indicated no significant difference between the USS and conventional groups in NTP (RR = 0.56, 95% CI [0.23, 1.33], p = 0.19) (Figure 11). The funnel plots of both subgroups were not applied because of the limited number of RCTs and nRCTs.

#### Abdominal drainage

**Figure 12:**
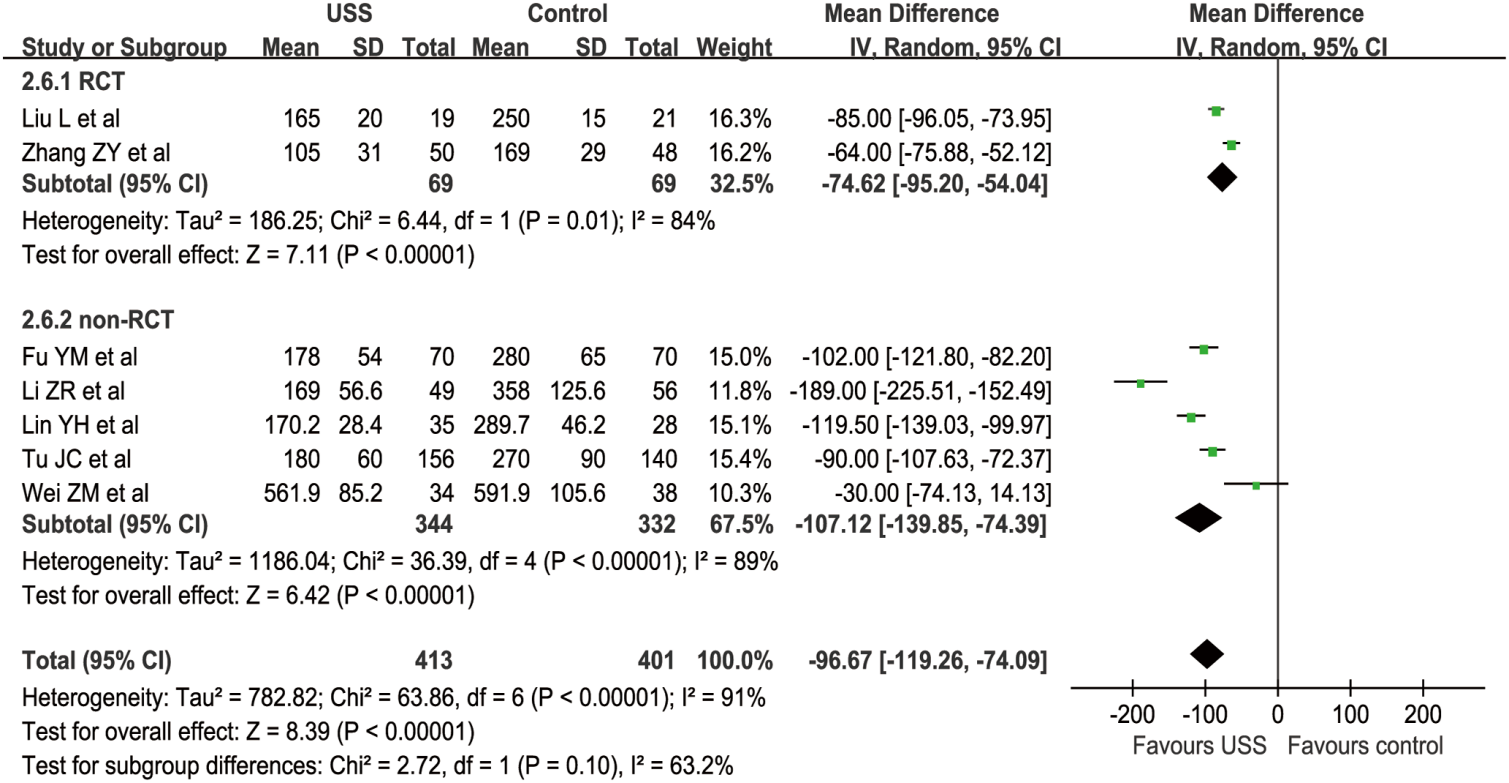
Forest plot of abdominal drainage.

**Figure 13:**
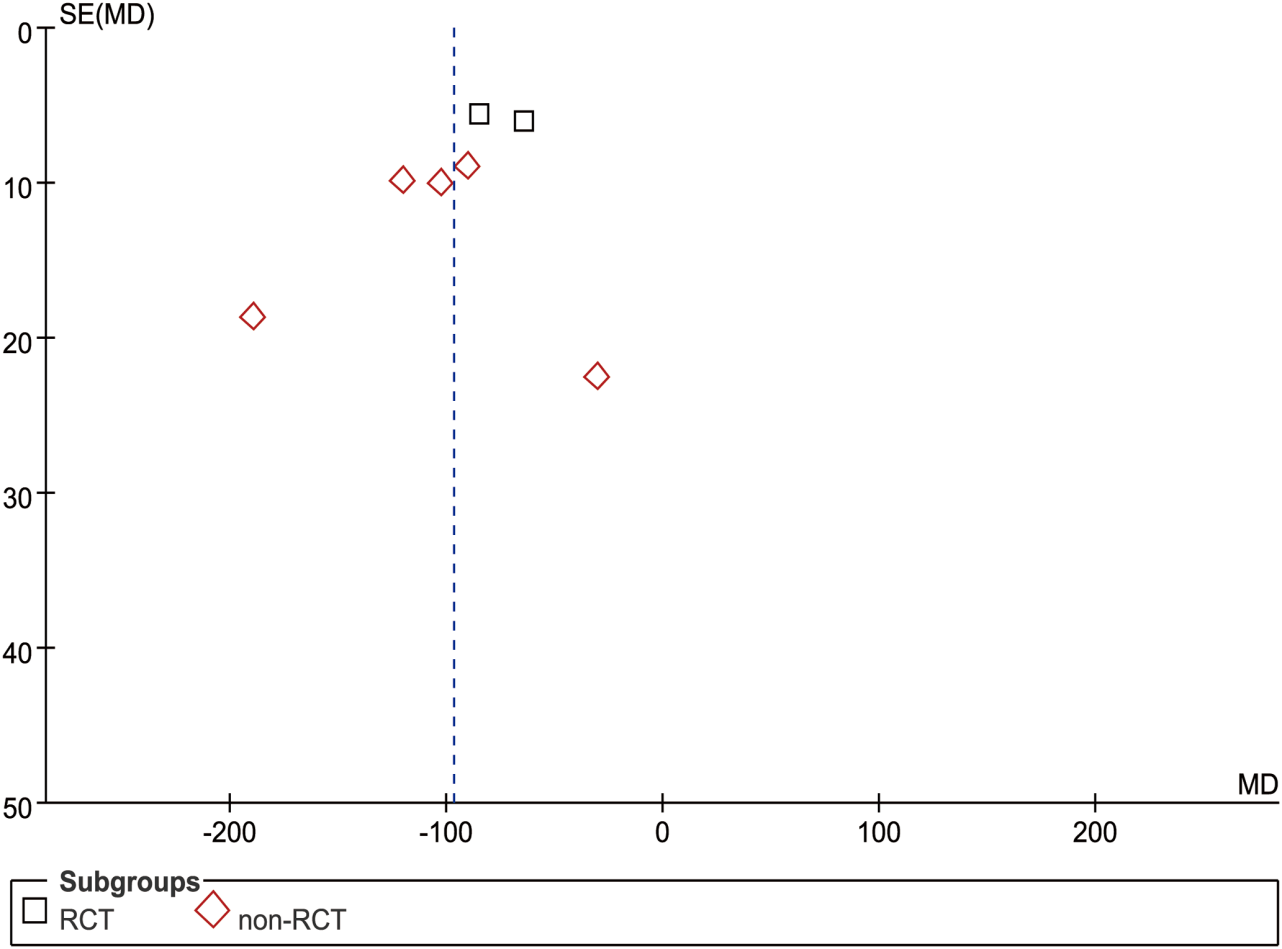
Funnel plot of abdominal drainage.

Seven studies (2 RCTs and 5 nRCTs) reported the total abdominal drainage within postoperative 3 days from 105 to 591.9 ml [22]–[24], [26]–[27], [32]–[33]. The WCM of the USS group was 199.2 ml compared with 302.8 ml in the control group. Meta-analysis showed that obviously less volume of abdominal drainage was found in the USS group than in the control group in both RCT (MD = −74.62, 95% CI [−95.20, −54.04], p<0.001) and nRCT subgroups (MD = −107.12, 95% CI [−139.85, −74.39], p<0.001). Taken together, patients in the USS group had approximately 100 ml drainage less than those in the conventional group within postoperative 3 days (MD = −96.67, 95% CI [−119.26, −74.09], p<0.001) (Figure 12). The funnel plot showed a symmetrical distribution of included studies (Figure 13).

#### Gastrointestinal function recovery days

**Figure 14:**
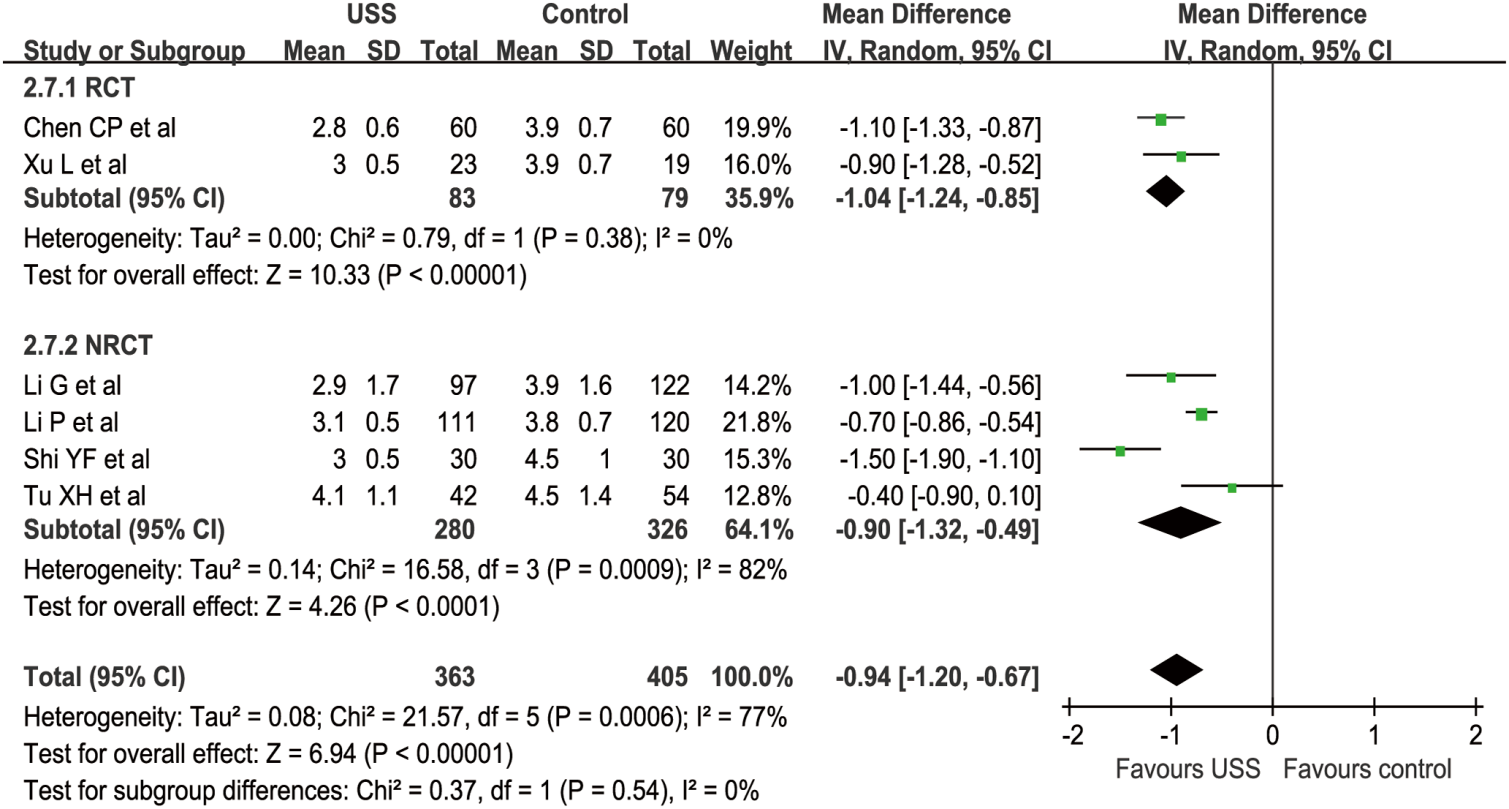
Forest plot of gastrointestinal function recovery days.

In our study, we considered both postoperative flatus and feeding as the indicator of the recovery of gastrointestinal function. In this regard, six studies (2 RCTs and 4 nRCTs) reported GIFRD from a mean of 2.8 to 4.5 days postoperatively [19], [25], [28]–[29], [34]–[35]. The WCM was 3.1 days in the USS group and 4.0 days in the control group. Meta-analysis demonstrated remarkably earlier recovery in the USS group than in the control group in both RCT (MD = −1.04, 95% CI [−1.24, −0.85], p<0.001) and nRCT subgroups (MD = −0.90, 95% CI [−1.32, −0.49], p<0.001). Generally, patients in the USS group reduced the recovery days by approximately one day compared with those in the conventional group (MD = −0.94, 95% CI [−1.20, −0.67], p<0.001) (Figure 14). The funnel plot was not applied because of the limited number of included studies.

## Discussion

This systematic review and meta-analysis compared the USS with conventional techniques regarding surgical efficacy and postoperative recovery in open gastrectomy. For surgical efficacy, meta-analysis showed significantly less OT and BL in USS operations than those in conventional ones. The advantage of USS in surgical efficacy might be attributed to the following four reasons: first, the USS is capable of finishing one-step cutting and coagulation procedures, meanwhile the pressure between USS clips can facilitate a hemostasis effect well; second, the vessels and lymphatics, of which, the pathways are intricate, are rich in perigastric tissues; third, many vessels and lymphatics should be cut off and coagulated if gastrectomy should be finished; fourth, various LNs are necessary to be dissected to prove the radical oncological effect of gastrectomy. For every cutting and coagulation, on the one hand, the USS could decrease the operation procedures and time compared with relative time-consuming tools, like monopolar electrocautery and thread ligation; on the other hand, satisfied hemostasis of vessels cut by the USS can decrease the times of thread ligation [37]. However, some vessels with large diameter (usually larger than 5 mm, such as left gastric vessels) had to be ligatured by threads or clips in case of hemorrhage [22]–[23], [27], [37]. Currently, neither USS nor monopolar electrocautery could cut off and coagulate large vessels well. In general, however, the USS showed superiority in surgical efficacy compared with conventional monopolar electrocautery.

For dissected LN, our meta-analysis indicated that more LNs could be dissected in the USS group than in the conventional group in the nRCT subgroup and overall effect analysis. Numerous LNs are usually known to be located closely along the vessels. Very delicate and precise operations are crucial if surgeons intend to finish dissection of LN without surgery-related injuries. Compared with conventional electric technology, the USS leads less adverse thermal injuries to the tissues adjacent to the target area. Besides, the USS has thin clips that can allow surgeons to divide tissues, cut off, and coagulate vessels conveniently in a relative narrow and deep space, as reported in other surgery [8]–[11]. Therefore, it might be easier and more secure for surgeons to dissect LN using USS than conventional methods.

Postoperative recovery was another important factor to estimate USS and conventional techniques. The POC rate is one of the most representative events for postoperative recovery. In this meta-analysis, complications of included studies were mainly graded as II-III according to the classification of surgical complications by Daniel Dindo et al [41]. More medical interventions and cost were likely to be involved when more complications occurred. Meanwhile, postoperative gastrointestinal function recovery, gastric tube decompression, and abdominal drainage are also indexes to assess postoperative recovery. Usually, gastrointestinal function recovery is mainly manifested through borborygmus, flatus, and feeding. In these included studies, flatus days, feeding days, and AD were reported more frequently. From our meta-analysis, there is no significant difference in POC between the USS and conventional groups in RCT and nRCT subgroups; however, significantly fewer POHD, less postoperative AD, and shorter GIFRD were found in the USS group than in the conventional group. Consequently, the USS could lead to relatively better and faster postoperative recovery, which we deduced benefited from the advantages of the USS in surgery as described previously.

In our study, the Jadad scale and NOS had been applied to evaluate the quality and potential bias of all included RCTs and nRCTs, respectively [15]–[16]. It showed that the scores of RCTs were located at the low level, mainly because of the absence of randomization details. The scores of nRCTs showed their possibility for meta-analysis. The published biases were also shown if the number of studies was suitable and enough for analysis. Additionally, the included studies were noted to be mostly from China, and the heterogeneity of some continuous variables was notable, although the random effect model was used. Because surgical skills of surgeons from different countries varied at different levels, there might be some extent of difference in OT, BL, and NDLN. Besides, the meta-analysis consisted of both RCTs and nRCTs. However, we performed the subgroup sensitivity analysis of every outcome classified by RCTs and nRCTs. Moreover, it was not in every estimated outcome that significant differences were simultaneously found in RCT and nRCT subgroup analysis. Because no significant differences were shown in NDLN (p = 0.88) and NTP (p = 0.19) in the RCT subgroup, we thought that the interpretation of these outcomes should be made conservatively. Another limitation of this study was that we only searched and analyzed published data and there was no gray literature that may usually indicate negative results. However, funnel plots were carried out in this study to assess the publication bias.

## Conclusion

Compared with conventional electrosurgery, the USS is a safe and effective technique with more short-term advantages in open surgery for gastric cancer, including shorter operation time, better hemostatic control and superior postoperative recovery.

## Supporting Information

Checklist S1 **PRISMA checklist.**
(DOC)

Flow Diagram S1 **PRISMA Flow Diagram.**
(DOC)
