# Supplementary material for: Comparison of Ultrasonic Scalpel versus Conventional Techniques in Open Gastrectomy for Gastric Carcinoma Patients: A Systematic Review and Meta-Analysis
Source: PLoS One. 2014 Jul 31;9(7):e103330. doi: 10.1371/journal.pone.0103330 (PMC4117513; doi:10.1371/journal.pone.0103330)
Supplement: Flow Diagram S1 — PRISMA Flow Diagram. (DOC) [file pone.0103330.s002.doc]

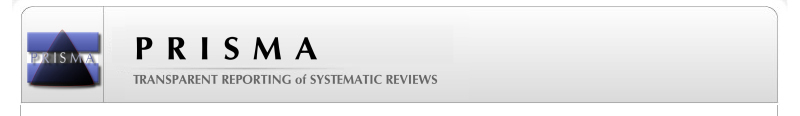
**PRISMA 2009 Flow Diagram**

**Screening**

**Included**

**Eligibility**

**Identification**

Records identified through CCRT, PubMed, EmBase and CNKI databases searching

(n = 673)

Records after duplicates removed

(n = 672)

Records screened

(n = 672)

Records without appropriate comparison like only ultrasonic scalpel (USS) group or records on other diseases like colon cancer were excluded
(n = 643)

Full-text articles assessed for eligibility
(n = 29)

Studies without clinical outcomes and appropriate comparison like USS group versus USS and conventional group were excluded.
(n = 10)

Studies included in qualitative synthesis
(n = 19)

Studies included in quantitative synthesis (meta-analysis)
(n = 19)

0 of additional records identified through other sources
